# MicroRNA-30a-3p: a potential noncoding RNA target for the treatment of arteriosclerosis obliterans

**DOI:** 10.18632/aging.205154

**Published:** 2023-10-27

**Authors:** Mao Zhang, Yu Chen, Fang Niu, Xiaohui Luo, Jiangping Li, Wei Hu

**Affiliations:** 1 Department of Vascular Surgery, Sichuan Provincial People’s Hospital, University of Electronic Science and Technology of China, Chengdu, China; 2 Department of Cardiology, Sichuan Provincial People’s Hospital, University of Electronic Science and Technology of China, Chengdu, China; 3 Department of Gynaecology and Obstetrics, Sichuan Provincial People’s Hospital, University of Electronic Science and Technology of China, Chengdu, China; 4 Department of Oncological Radiotherapy, Cancer Center and State Key Laboratory of Biotherapy, West China Hospital, Sichuan University, Chengdu, China

**Keywords:** cardiovascular disease, peripheral arterial disease, arteriosclerosis obliterans, atherosclerosis, miRNA

## Abstract

An increasing number of studies have shown that noncoding RNAs are involved in cardiovascular diseases. Our study shows that the expression of microRNA-30a-3p (miR-30a-3p) in patients with arteriosclerosis obliterans (ASO) of the lower extremities is significantly decreased after endovascular treatment, but its role is unclear. This study aims to explore the role of microRNA-30a-3p in ASO and its related mechanisms. Immunofluorescence and *in situ* hybridization costaining indicated that microRNA-30a-3p mostly exists in vascular smooth muscle cells (VSMCs). Furthermore, after transfection into VSMCs, microRNA-30a-3p inhibited VSMC proliferation, migration and phenotype switching. In addition, luciferase reporter and western blot analyses indicated that ROCK2 (Rho-related spiral coil 2 containing protein kinase) is a microRNA-30a-3p target gene, and participates in the microRNA-30a-3p mediated cell inhibitory effect. At last, the rat carotid artery was infected by lentivirus after balloon injury, which increased microRNA-30a-3p levels and apparently suppressed the formation of neointima *in vivo*. Overall, exogenous introduction of microRNA-30a-3p, a noncoding RNA with unlimited potential, may be a new approach to treat ASO.

## INTRODUCTION

Cardiovascular disease treatments have developed rapidly in recent decades, but vascular diseases have become a worldwide health problem and lead to serious heart disease, as well as serious vascular diseases in many other parts of the body, such as vascular dementia, stroke, lower limb arteriosclerosis obliterans (ASO), diabetic retinopathy, and diabetic nephropathy [1, 2]. Among these conditions, lower extremity ASO has a high incidence and is a serious disease and one of the main causes of limb loss [3]. A series of endovascular procedures, such as plaque rotation, stent implantation and balloon angioplasty, are important treatments for lower extremity ASO, but postoperative vascular restenosis seriously hinders the recanalization of ASO vessels in the lower limbs [4]. Therefore, identification of a method that can inhibit arterial stenosis is urgently needed.

Whether vascular restenosis occurs after endovascular treatment or the occurrence and development of ASO, its main pathological manifestations are platelet aggregation, lipid deposition, inflammatory cell invasion, growth factor stimulation, vascular smooth muscle cell (VSMC) migration and proliferation, extracellular matrix regeneration, etc., and traditionally, the excessive VSMC proliferation and migration after external stimulation is the most important reason among these manifestations [4]. Therefore, exploring the mechanism underlying VSMC function might help elucidate the pathogenesis and identify a new therapeutic strategy.

In recent decades, major developments in the field of basic research have been reported, and new discoveries in the field of noncoding RNA are emerging, which have led to new paradigms in the treatment of diseases. Some noncoding RNAs, such as microRNAs, lncRNAs, circRNAs, piRNAs, rRNAs and tRNAs, have been identified in the past few decades, and have been shown to be biomarkers and therapeutic targets for cardiovascular diseases by numerous studies [5, 6]. Among them, microRNAs are endogenous, single-stranded, small (19 to 23 nucleotides) noncoding RNAs that exist in almost all organisms and have been suggested to be closely related to gene regulation; therefore, they are involved in the occurrence and development of ASO [7]. MicroRNAs mediate their effects mainly by translational inhibition or RNA cleavage/degradation to negatively regulate target gene function. For example, miR-4463 can promote VSMC phenotypic switching by targeting bFGF8 [8]. Based on our existing research results, microRNA-30a-3p was notably reduced in restenotic ASO vessels after endovascular treatment, which attracted our interest.

Our study investigates the possible role of microRNA-30a-3p in cardiovascular disease for the first time. The experimental results may provide new potential biomarkers and novel noncoding RNA-based approaches for ASO diagnosis and treatment.

## MATERIALS AND METHODS

### Sample acquisition

Vascular specimens were obtained from donors with or without arteriosclerosis obliterans who underwent lower limb amputation since 2014. These specimens were approved by donors or by their families. Patient information is in Supplementary Table 1.

### Real-time qRT-PCR

First, we degraded the vascular tissue or cell and extracted the RNA. Then, the PrimeScriptTM RT kit (TaKaRa, Japan) was used to generate total cDNA. Next, we used a real-time PCR apparatus (ABI, USA) and SYBR Green PCR Kit (TaKaRa, Japan) for qPCR detection. Table 1 shows the primer sequences for qRT-PCR.

**Table 1:** Sequences of the primers used for qRT-PCR.

**Name**	**Forward**	**Reverse**
**GAPDH**	tgacttcaacagcgacaccca	caccctgttgctgtagccaaa
**ROCK2**	ctttcattgcctgtacgaaaca	actggtcggacatgaaataact
**MiR-30a-3p**	gctcgtcctttcagtcggatgt	tccagtgcagggtccgagg
**U6**	ctcgcttcggcagcaca	aacgcttcacgaatttgcgt

### Cell culture

VSMCs were prepared by the explant method, and anti-alpha SMA antibody (ab5694, Abcam, UK) staining was used for identification. The cell suspension was assessed with a counting plate. The number of cells was adjusted to (2-5) × 10^5^ cells/ml, or the density required for the experiment. VSMCs were placed in a 37° C incubator containing 5% carbon dioxide. The Dulbecco’s modified Eagle’s medium (DMEM) (Invitrogen, USA) containing 5% serum and 1% penicillin and streptomycin was used. In general, the primary cultured cells (Supplementary Figure 2) adhered to the bottle wall and began to grow after 5-7 days. New medium containing 1/2 of the original medium was added, and the medium was changed after 2-3 days for further culture. Generally, after 7-14 days, cells filled the bottle wall for generations.

### Cell transfection and infection

Smooth muscle cells were seeded at 3×105 cells/well into 6-well plate. After 24 hr, cells were transfected with microRNA-30a-3p analog, inhibitor and control oligos (RiboBio, China), respectively using LipofectamineTM 3000 reagent (Invitrogen, USA). For lentiviral transfection, ROCK2-LV (Gene Copoeia, USA) lentivirus particles were utilized according to the instructions.

### *In situ* hybridization

We used the 5’DIG-labeled LNA probe (Supplementary Table 2) (TaKaRa, Japan) and miRCURY LNA™ Detection Control Probe (Exiqon, Denmark) for *in situ* hybridization to detect microRNA-30a-3p in 5 μm paraffin sections. Then, we used a tyramine signal amplification system (PerkinElmer, USA) to amplify the localization signal of microRNA-30a-3p according to the instructions. Finally, VSMCs were stained with muscle-α-actin antibodies. ImageJ was used to analyze the section’s photos.

### CCK-8 assay

The treated VSMCs were plated at 1×103 cells/well into 96-well plate in five replicates. Cell proliferation was measured using CCK-8 kit (Dojindo, Japan), and then the relative smooth muscle cell proliferation rates were measured by measuring absorbance values at 450 nm wavelength.

### EdU assay

We used an EdU Kit (RiboBio, China) to conduct EdU experiments in treated or transfected VSMCs. In the EdU analysis process, 100 μL EdU was first used to treat VSMCs in 96-well culture plates for 2 hours, and Hoechst 33342 staining was then used to observe the nucleus. Finally, positive cells were counted and normalized to the total number of cells (positive rate = positive cell / total cell).

### Transwell assay

Treated or transfected cells were placed in the upper well (Costa, USA), serum-free medium was used, and medium with serum was placed in the lower well. After 12 hours, the Transwell chambers were fixed, and we then stained smooth muscle cells with crystal violet dye to assess cell migration ability [9]. Cells in five random fields were selected for counting.

### Wound healing assay

For the wound healing assay, following the transfection, the VSMCs were seeded into 6-well plates (30,000 cells/well) until 90% confluence was reached, and then, a single scratch wound was produced using a sterilized 200-μL disposable pipette tip. The cells were cultured with medium without serum for 0 to 24 hours, scratch wounds were visualized using an inverted microscope, and the area of the scratch wounds was measured using Image Pro Plus software. The wound-healing rate demonstrates the percentage of wound healing with the initial scratch area as 100%, and the wound healing rate was compared between groups with the vehicle used in the control group.

### Luciferase reporter assay

First, by designing primers to clone desired target fragments from genomic DNA by PCR, we generated wild-type or mutant-type sequences of the ROCK2 3’-untranslated region (UTR). Next, the sequences were added to the psi-CHECK2TM vector to construct plasmids (sequence information of the target genes for luciferase reports is in Supplementary Table 3 and Supplementary Figure 1). Then, we cotransfected the microRNA-30a-3p mimic, inhibitor, blank control and constructed plasmids into HEK298 cells by Lipofectamine for further detection. Two days later, the instructions of the Luciferase Reporter Kit (Promega, USA) were followed to detect the luciferase activity. Finally, R-luc activity was compared with control F-luc activity to determine transfection efficiency.

### Western blot assay

We first extracted the total protein from the lysate of cells or tissues and then separated the protein by electrophoresis. Then the proteins on the gels were transferred onto PVDF membranes. We used the antibodies listed below to measure the relative expression of the following proteins: SM-α-actin (AB5694, Abcam, UK), ROCK2 (21645-1-AP, Proteintech, China), GAPDH (A00084, GenScript, China), calponin (AB46794, Abcam, UK), and Ki67 (28074-1-AP, Proteintech, China). Finally, enhanced chemiluminescence (ECL) detection reagent (Applygen Technologies, China) was added to the PVDF film (Millipore, USA), and then, photos were taken.

### Rat carotid artery injury model

First, we clamped the unilateral arteria carotis communis (as close to the heart as possible) and the arteria carotis interna and then ligated the arteriae carotis externa near the cerebral artery. Then, the arteriae carotis externa was cut locally, and this incision did not exceed one-third of the vessel perimeter. A 2F Forgarty balloon was then inserted retrogradely into the arteria carotis communis and inflated, and we monitored the size of the balloon to more easily prop up the vessel. The balloon was pumped back and forth (3-5 times) to remove the vascular endothelium. The balloon device was carefully withdrawn, and then we added the virus transfection solution into the temporary arteria carotis communis occluded site, waiting for 10 minutes for infection. Finally, we ligated the arteriae carotis externa and closed the surgical incision. Two weeks later, these rats were sacrificed, and the arteria carotis were removed for analysis.

### Statistics

Student’s t test was selected to analyze the data, and a *p*-value < 0.05 was considered statistically significant. In these experiments, we analyzed the data with GraphPad Prism.

## RESULTS

### MicroRNA-30a-3p decreased in the restenotic ASO artery

**Figure 1:**
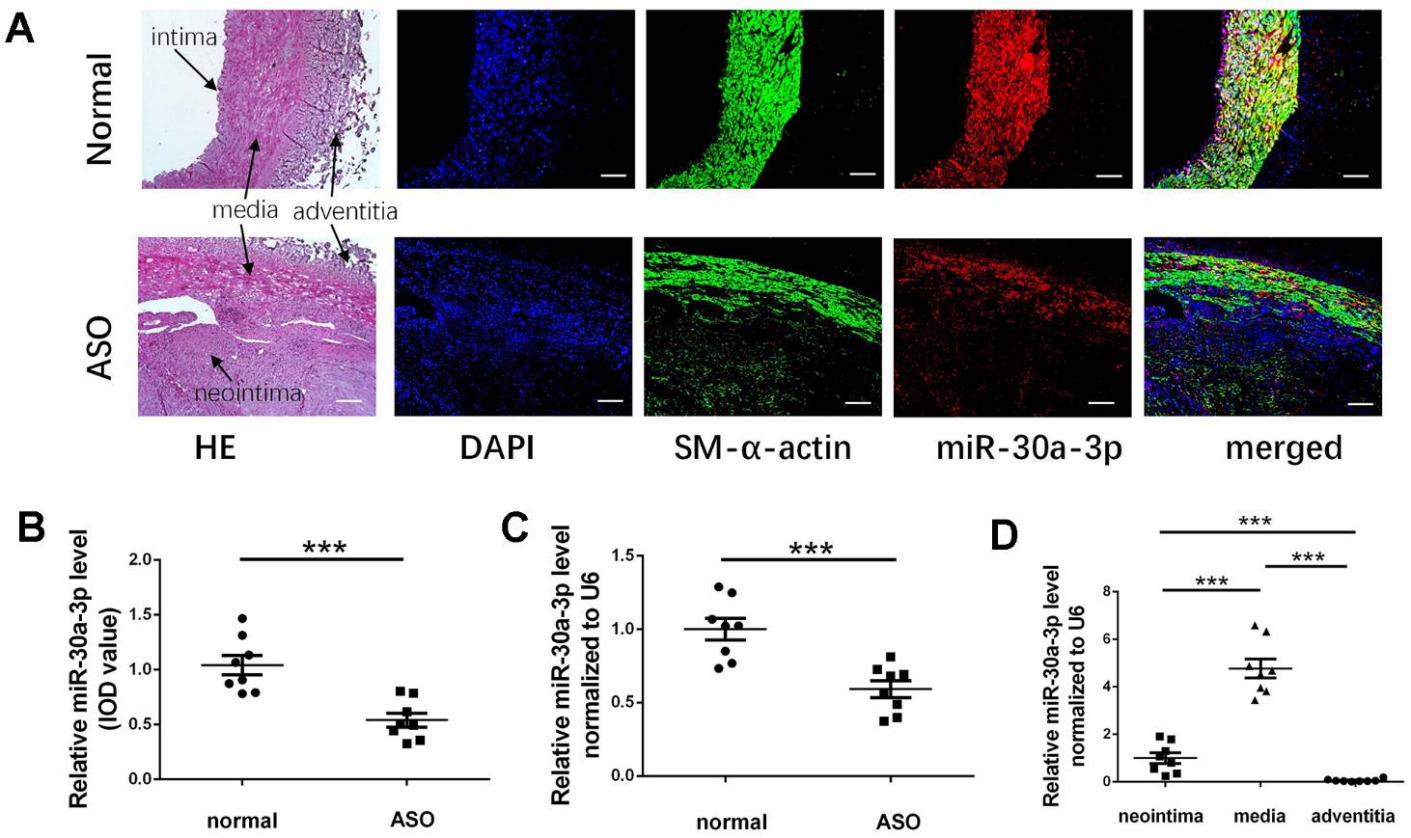
**MicroRNA-30a-3p decreased in the restenotic ASO artery.** (**A**) SM-α-actin and microRNA-30a-3p were stained in ASO and normal vascular sections (n=8); We used DAPI to stain the nuclei. (**A**, **B**) The IOD of the microRNA-30a-3p-stained vascular sections was calculated and compared. (**C**) Relative microRNA-30a-3p levels in ASO and normal arteries (qRT-PCR, n=8). (**D**) Relative microRNA-30a-3p levels in the three layers of ASO arteries (qRT-PCR, n=8). Scale, 200 μm; ***=0.001.

In ASO arteries with restenosis after endovascular treatment, the distribution ranges of microRNA-30a-3p and the VSMC marker SM-α-actin were basically consistent, as determined by immunofluorescence and *in situ* hybridization analysis (Figure 1A). According to this result, we believe that the microRNA-30a-3p was concentrated in VSMCs (Figure 1A). Then, the IOD (integrated optical density) value of these fluorescently stained sections was detected and compared. By analyzing the IOD value, we found that microRNA-30a-3p expression in ASO arteries was markedly lower than that in normal arteries (Figure 1B). Beyond that, by qRT-PCR analysis also showed that microRNA-30a-3p levels in ASO arteries were decreased (Figure 1C). Combined staining results also revealed that in ASO vessels, the microRNA-30a-3p content in neointima was markedly lower than that in the central smooth muscle layer. To further confirm this result, we separated the three layers (neointima, media, adventitia) of the ASO artery sample with forceps and then detected the relative miRNA levels. The content of micoRNA-30a-3p in the middle layer was higher than that in the other two parts (Figure 1D). Finally, in summary, microRNA-30a-3p in the ASO smooth muscle layer is significantly reduced, especially in the neointima.

### The VSMC function was suppressed by MicroRNA-30a-3p

**Figure 2:**
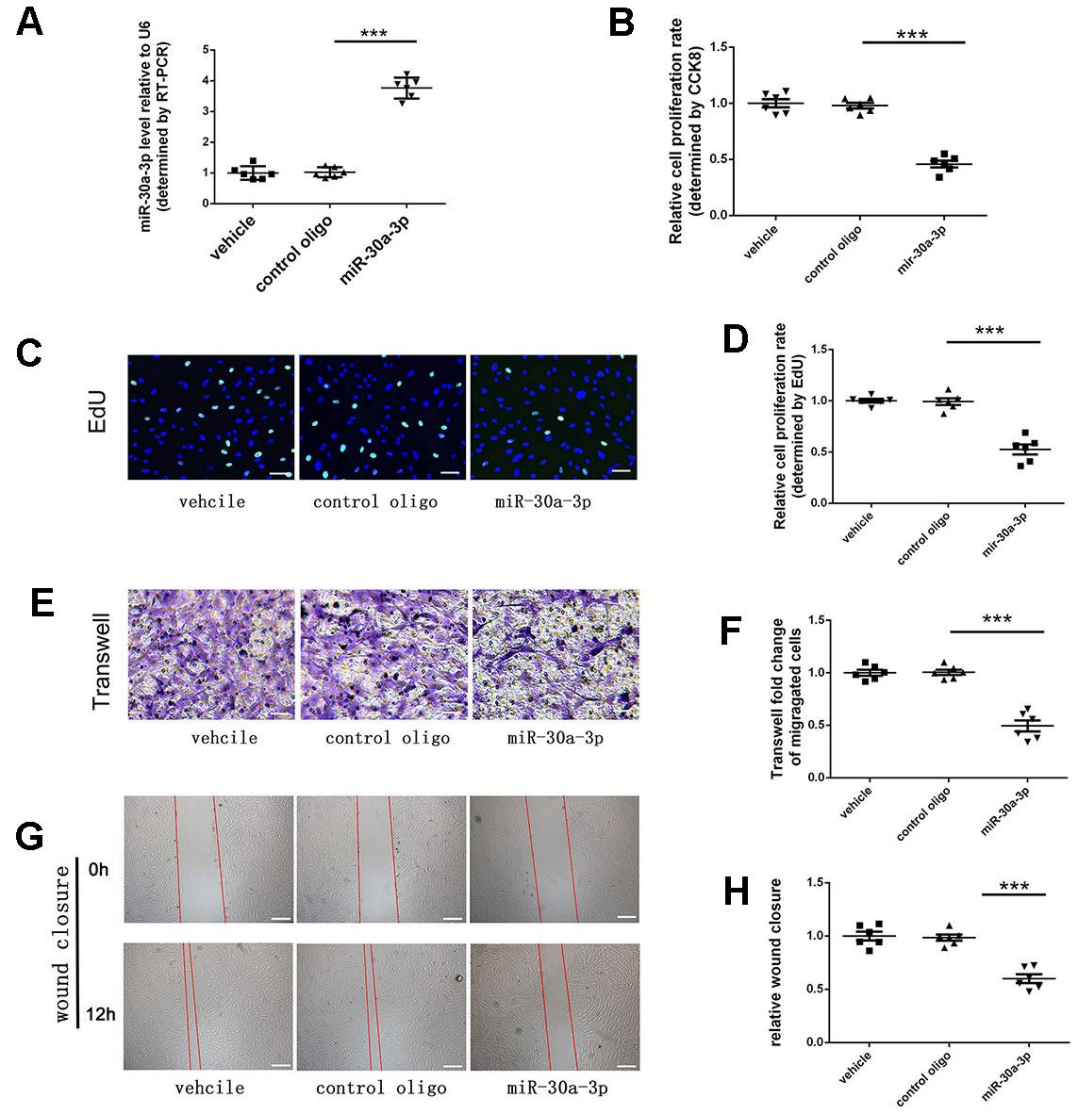
**VSMC proliferation and migration were suppressed by microRNA-30a-3p.** (**A**) MicroRNA-30a-3p levels in each group (RT-PCR, n=6). MicroRNA-30a-3p suppressed VSMC proliferation, as shown by (**B**) CCK-8 assays (n=6) and (**C**, **D**) EdU assays (n=6). MicroRNA-30a-3p suppressed VSMC migration, as shown by (**E**, **F**) Transwell assays (n=6) and (**G**, **H**) wound healing assays (n=6). Scale, 100 μm (**C**, **E**), 200 μm (**G**); ***=0.001.

MicroRNA-30a-3p mimics were first transfected into human VSMCs and then, through qRT-PCR detection, the content of microRNA-30a-3p in VMSCs was found to be markedly increased (Figure 2A). Furthermore, upregulation of microRNA-30a-3p content in VSMCs was confirmed to reduce the proliferative capacity of VSMCs by both experiments with CCK-8 (Figure 2B) and Edu (Figure 2C, 2D) assays. Next, we also found that when the microRNA-30a-3p content in cells increased, VSMC migration capacity *in vitro* was inhibited in two experiments, Transwell assays (Figure 2E, 2F) and wound healing analysis (Figure 2G, 2H). In summary, we can conclude from these experiments that VSMC proliferation and migration were suppressed by microRNA-30a-3p.

### The phenotypic switch of VSMCs was suppressed by MicroRNA-30a-3p

**Figure 3:**
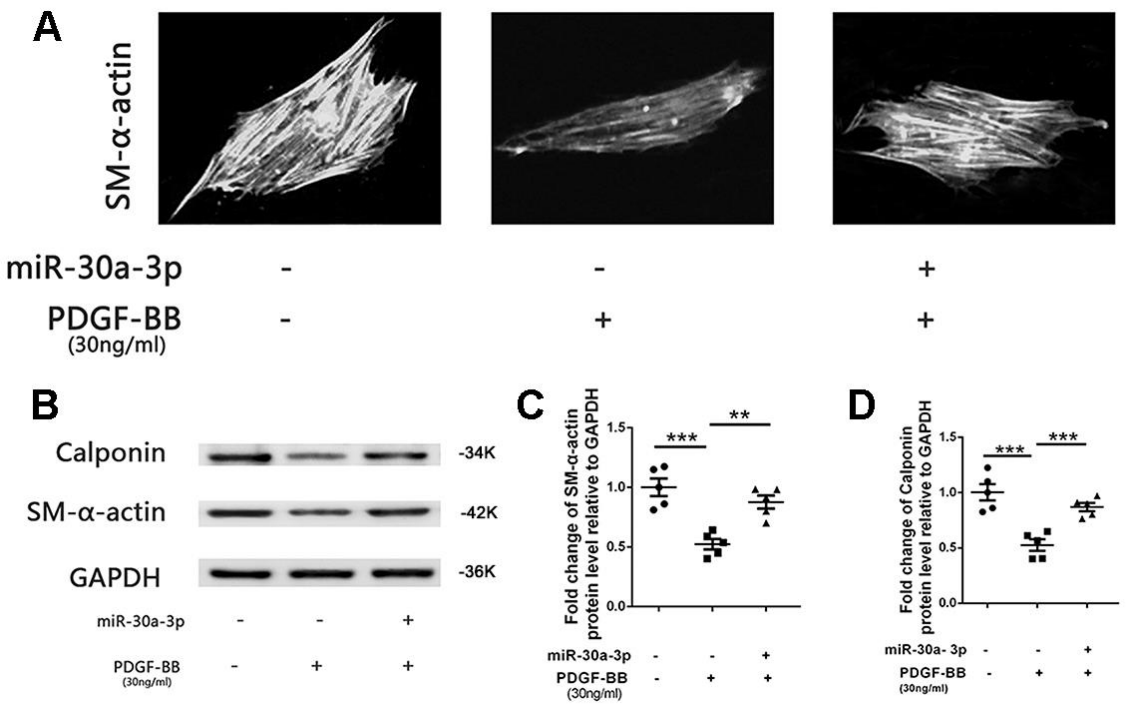
**The VSMC phenotypic switch was suppressed by microRNA-30a-3p.** (**A**) PDGF-BB stimulation promoted VSMC phenotypic switching from contractile conditions to synthetic conditions, and microRNA-30a-3p upregulation suppressed this process. (**B**–**D**) MicroRNA-30a-3p suppressed VSMC phenotypic protein (SM-α-actin and calponin) switching. **=0.005, ***=0.001.

The VSMCs phenotype can change from contractile to synthetic after stimulation by PDGF-BB. In this study, we examined whether this phenotypic switch of VSMCs is mediated by microRNA-30a-3p. The experiment confirmed that microRNA-30a-3p upregulation suppressed the VSMC phenotypic switch induced by PDGF-BB (Figure 3A). In general, the phenotype protein expression of SM-α-actin and calponin was decreased during PDGF-BB-stimulated VSMC switching. Further studies indicated that excess microRNA-30a-3p suppressed the VSMC phenotypic switch process, and the content of related phenotypic proteins was partially restored (Figure 3B–3D).

### MicroRNA-30a-3p directly targets ROCK2

Through three miRNA target prediction software programs (RNA22, TargetScan, miRDB), we screened 859 possible microRNA-30a-3p target genes, and these three programs predicted these target genes at the same time. By using DAVID (an annotation, visualization and comprehensive discovery database) to annotate GO functions and enrich the KEGG pathways of these 859 genes, we found that 52 genes regulate cell migration and proliferation and 16 genes regulate the actin cytoskeleton, which may be involved in the phenotypic switch of VSMCs. There were 5 genes belonging to both groups of genes: APC, KRAS, ROCK2, FGF5, and ITGA5. Among these genes, ROCK2 has been widely studied in arteriosclerosis and vascular smooth muscle cells [10–12], indicating that in ASO, ROCK2 may be involved in the microRNA-30a-3p functional process and may be a microRNA-30a-3p direct target gene.

**Figure 4:**
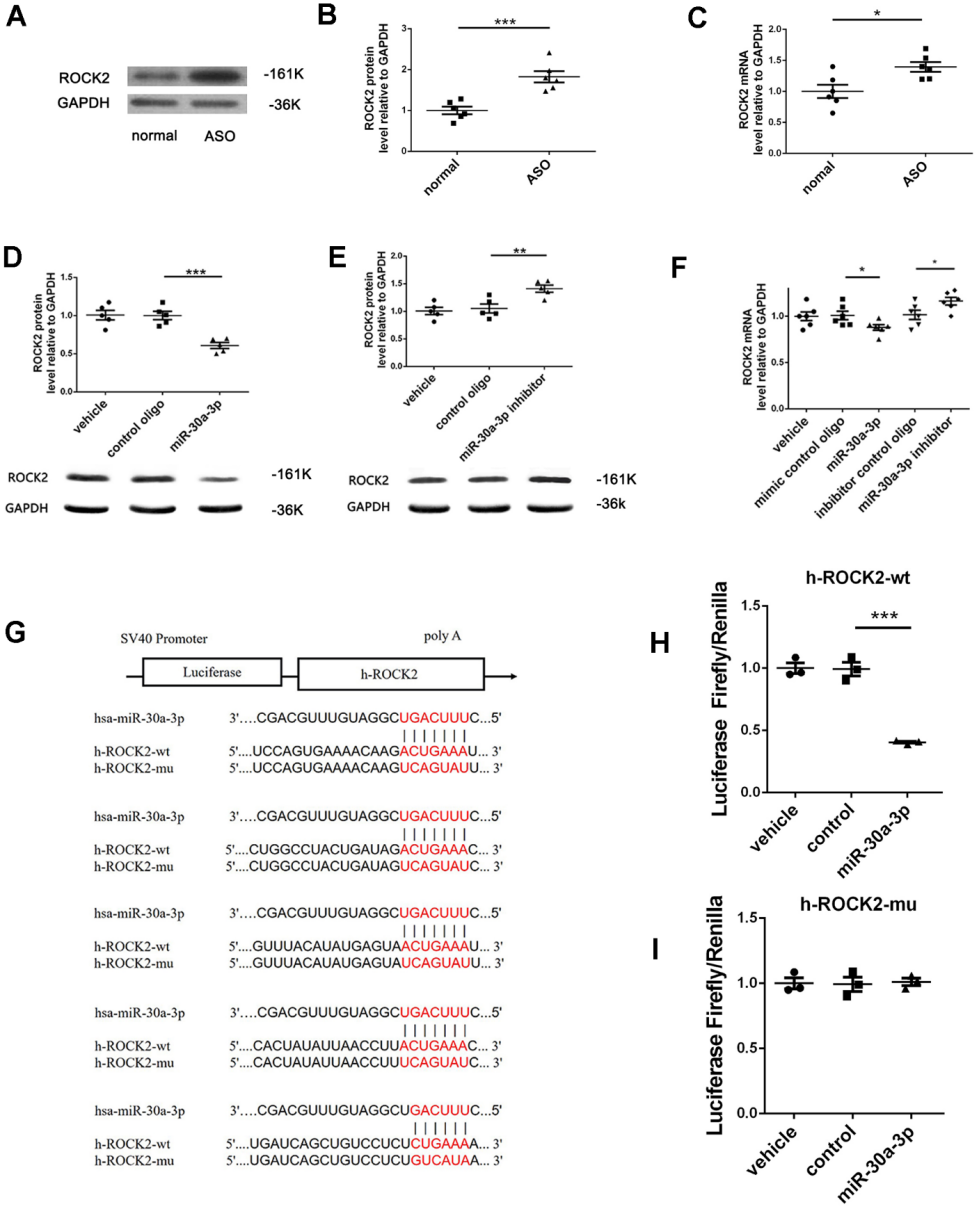
**MicroRNA-30a-3p targeted ROCK2.** (**A**, **B**) ROCK2 protein levels in ASO vasculature were higher than those in normal vessels (WB, n=6). (**C**) ROCK2 mRNA levels in ASO vasculature were greater than those in normal tissues (qRT-PCR, n=6). (**D**, **E**) MicroRNA-30a-3p downregulated ROCK2 protein expression, and the microRNA-30a-3p inhibitor upregulated ROCK2 protein expression in VSMCs (WB, n=5). (**F**) The microRNA-30a-3p mimic downregulated ROCK2 mRNA expression, and the microRNA-30a-3p inhibitor minimally upregulated ROCK2 mRNA expression in VSMCs (qRT-PCR, n=6). (**G**) The ROCK2 3’-UTR has five microRNA-30a-3p binding sites, and we synthesized wild-type and mutant-type luciferase reporter vectors: h-ROCK2-wt and h-ROCK2-mu. (**H**) Coinfection with microRNA-30a-3p and wild-type h-ROCK2-wt significantly decreased the luciferase activity; (**I**) however, coinfection with microRNA-30a-3p and mutated h-ROCK2-mu did not affect the luciferase activity (n=3). *=0.05, **=0.005, ***=0.001.

Through western blot assays, we found that the arterial tissue of the ASO group had higher ROCK2 protein content than normal tissue (Figure 4A, 4B), whereas in contrast, microRNA-30a-3p levels were decreased (Figure 1A). In addition, we measured ROCK2 mRNA level in ASO arterial tissue, which was also higher than that in the normal control (Figure 4C). The experimental results indicate an inverse correlation between microRNA-30a-3p and ROCK2.

For further research, we transfected VSMCs with microRNA-30a-3p or inhibitor to observe their effects on target genes. We detected changes in ROCK2 protein and RNA levels to verify whether ROCK2 is a microRNA-30a-3p target gene. The result was consistent with our expectations; microRNA-30a-3p reduced ROCK2 protein levels (Figure 4D, 4E) and mRNA levels (Figure 4F) in VSMCs, whereas the microRNA-30a-3p inhibitor increased their levels.

Through different databases, we found five potential microRNA-30a-3p targets in the ROCK2 3’-UTR, which are conserved in human, rat and mouse species. Thus, ROCK2 is a possible microRNA-30a-3p target gene. Then, a luciferase reporter assay indicated that microRNA-30a-3p could target the ROCK2 3’-UTR. First, we cloned the ROCK2 mRNA 3’-UTR sequence including these five possible microRNA-30a-3p target sites into an empty vector of the dual luciferase reporter (Figure 4G). As shown in Figure 4H, in the wild-type ROCK2 group, microRNA-30a-3p inhibited the relative luciferase activity, while in the mut-type ROCK2 group, the microRNA-30a-3p inhibitory effect on luciferase activity was weakened (Figure 4I). The experiment indicated that ROCK2 is indeed a microRNA-30a-3p target gene.

### ROCK2 attenuated the function of MicroRNA-30a-3p

**Figure 5:**
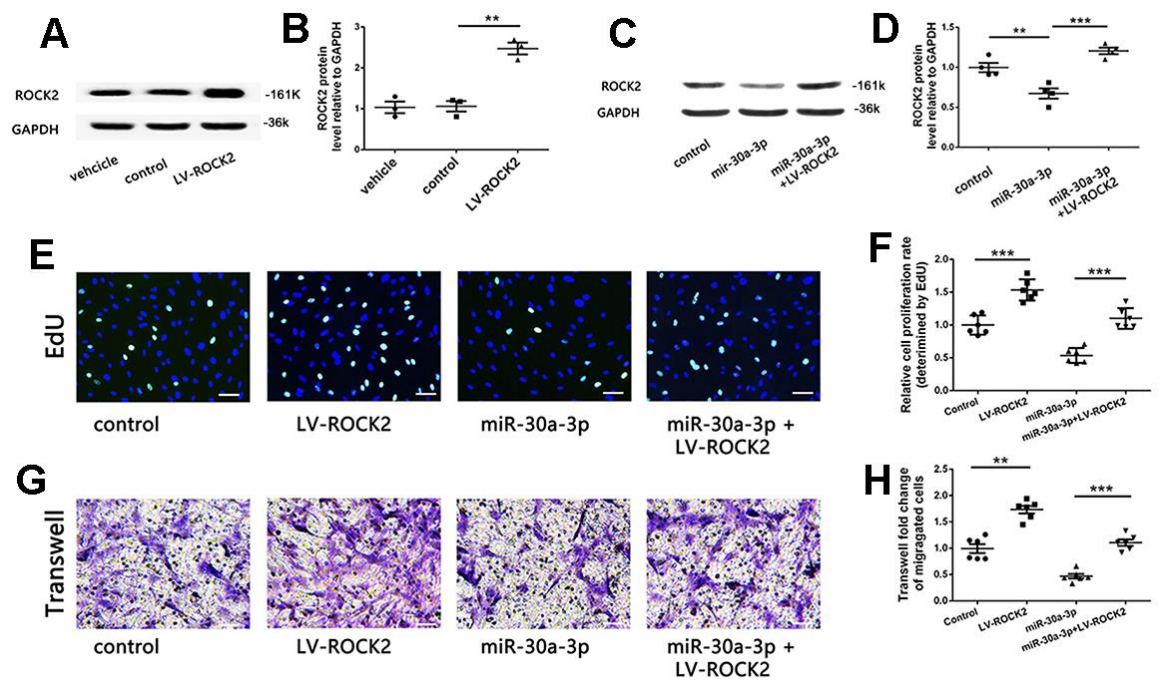
**ROCK2 attenuated the microRNA-30a-3p inhibitory effect.** (**A**, **B**) A lentivirus vector (LV-ROCK2) upregulated ROCK2 in VSMCs (WB, n=3). (**C**, **D**) LV-ROCK2 infection recovered ROCK2 expression levels in microRNA-30a-3p-transfected VSMCs (WB, n=6). (**E**, **F**) LV-ROCK2 promoted VSMC proliferation and attenuated the inhibition of VSMC proliferation by microRNA-30a-3p (EdU assay, n=6). (**G**, **H**) LV-ROCK2 promoted VSMC migration and attenuated the microRNA-30a-3p inhibitory effect on VSMC migration (Transwell, n=6). Scale, 100 μm (**E**, **G**); *=0.05, **=0.005, ***=0.001.

Experiments have shown that microRNA-30a-3p directly targets ROCK2. Moreover, ROCK2 protein content was decreased, and smooth muscle cell function was also inhibited by microRNA-30a-3p. Therefore, if we reincreased the protein content of ROCK2 in microRNA-30a-3p-transfected VSMCs, we should be able to rescue the microRNA-30a-3p inhibitory effects on VSMCs. Therefore, we first constructed a ROCK2 lentivirus vector containing only the CDS region of ROCK2 but not the 3’-UTR and then infected VSMCs. LV-ROCK2 increased the level of ROCK2 in VSMCs (Figure 5A, 5B), and LV-ROCK2 counteracted microRNA-30a-3p inhibition of ROCK2 (Figure 5C, 5D). In the next experiment, we found that upregulation of ROCK2 improved VSMC proliferation ability after transfection with microRNA-30a-3p (Figure 5E, 5F) and also migration ability (Figure 5G, 5H). Therefore, the effects of microRNA-30a-3p in VSMC proliferation and migration can be effectively resisted by upregulating ROCK2 expression. In summary, the above experiments show that microRNA-30a-3p directly targets ROCK2 and realizes its own functions through ROCK2.

### MicroRNA-30a-3p inhibited neointima formation

**Figure 6:**
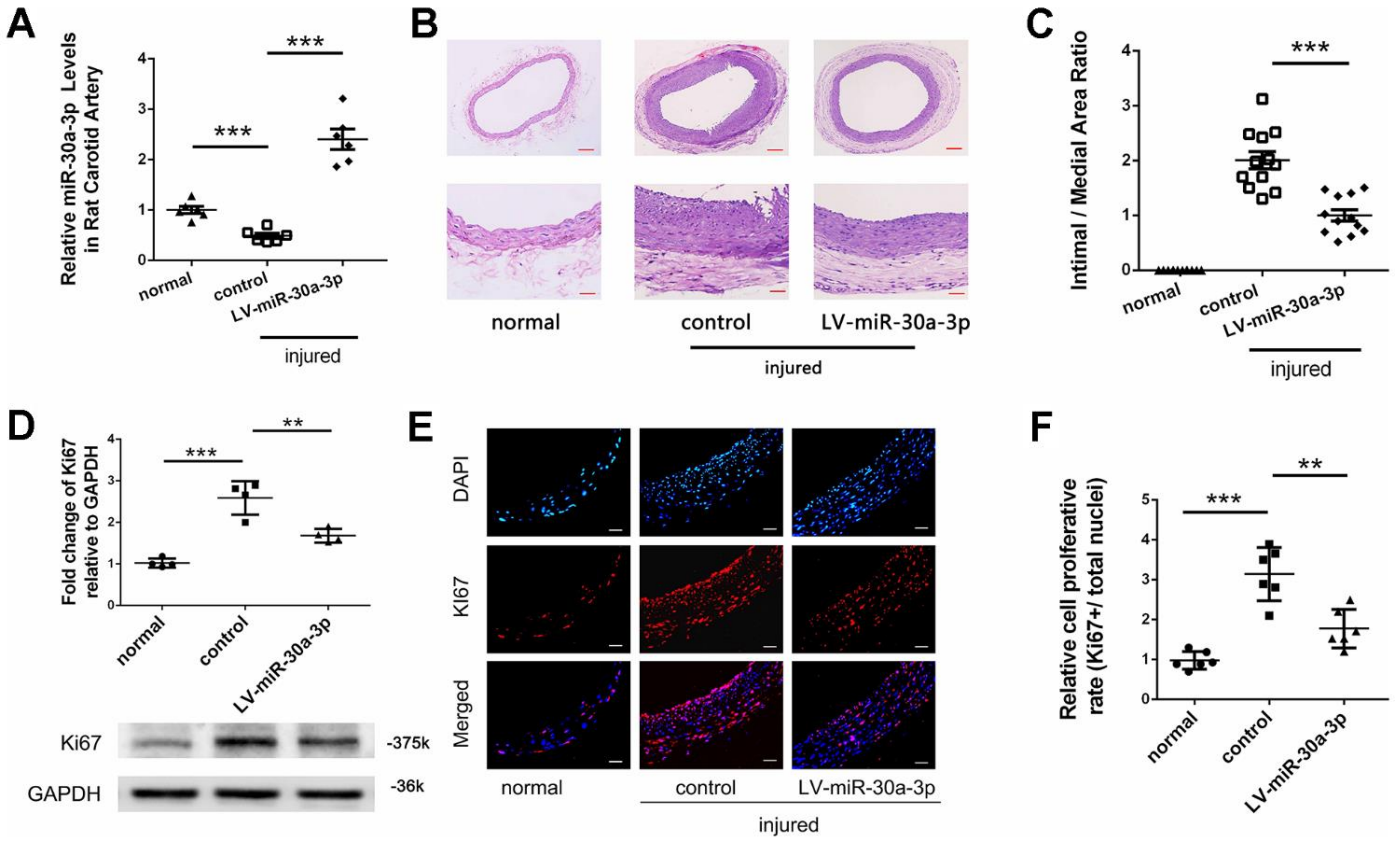
**MicroRNA-30a-3p inhibited neointima formation.** (**A**) LV-miR-30a-3p increased microRNA-30a-3p levels in the balloon injury model (qRT-PCR, n=6). (**B**, **C**) Neointima formation was inhibited by LV-miR-30a-3p (n=12). LV-miR-30a-3p suppressed VSMC proliferation as shown by the Ki67 analysis (**D**) (WB, n=4) and (**E**, **F**) (IF, n=6). Scale, 50 μm (bottom of **B**, **E**), 200 μm (top of **B**); **=0.005, ***=0.001.

We used SD rats to construct a carotid artery injury model and used this model to explore whether microRNA-30a-3p interferes with neointimal formation. First, we upregulated microRNA-30a-3p expression in carotid artery tissue of an animal model by using a lentivirus vector. In the model, the microRNA-30a-3p level in vascular tissue was originally decreased, which is consistent with the change in human ASO arterial tissue after endovascular treatment. Then, we infected vascular tissue with a lentivirus vector (LV-miR-30a-3p) to upregulate microRNA-30a-3p expression, and the results were verified by qRT-PCR detection (Figure 6A). The following tests indicated that LV-miR-30a-3p suppressed neointima formation in the model (Figure 6B, 6C). Moreover, Ki67 protein, a marker of cell proliferation, was detected by western blotting (Figure 6D) and immunofluorescence (Figure 6E, 6F). These Ki67 changes showed that LV-miR-30a-3p suppressed the VSMC proliferation rate in the model. The above experiments proved that exogenous introduction of microRNA-30a-3p into vascular tissue can significantly reduce the formation of neointima.

## DISCUSSION

Cardiovascular disease has become an important public health problem worldwide. Among arteriosclerotic vascular diseases, the incidence rate of lower limb ASO is higher, second only to coronary heart disease and stroke [13]. Drug and exercise therapy has been unable to effectively address this condition, and endovascular intervention has become an important therapeutic method. In recent decades, with the rapid development of endovascular therapy, matching tools have also been greatly developed, including drug elution technology, special balloons and bionic stents [14]. Of course, open bypass surgery is still an important choice for advanced diseases. However, the issue of restenosis remains and can lead to severe consequences such as limb amputation. We look forward to newer approaches that complement or even replace current therapies.

In the past few decades, an increasing number of studies have shown that noncoding RNAs (miRNAs, lncRNAs, circRNAs, piRNAs, rRNAs, tRNAs and other small RNAs) play various roles in cardiovascular disease occurrence and progression [15]. With the rapid development of the field of noncoding RNA research, researchers have developed increasingly advanced methods to regulate these noncoding RNAs to establish a battlefield against cardiovascular disease in new strategies. MicroRNAs, as noncoding RNAs, play important roles in the treatment of cardiovascular diseases and are the most commonly studied noncoding RNAs [15].

The development of arteriosclerosis is closely related to the abnormal expression of microRNAs. Many experiments have shown that microRNAs are important factors in VSMC function [16]. Excessive VSMC proliferation and migration exist in at all stages of arteriosclerosis development [17]. Through early chip screening, we found that miRNA-30a-3p had a strong influence on VSMC function by the screening of dozens of miRNAs with differential expression; thus, we focused on miRNA-30a-3p. Based on this experiment, we discovered that microRNA-30a-3p was distributed in VSMC of vascular tissue and confirmed that its expression in restenotic ASO arteries was decreased. Which indicates microRNA-30a-3p involvement in the pathogenesis of lower extremity ASO after endovascular treatment, which aroused our interest. In studies related to arteriosclerosis, we examined microRNA-30a-3p in VSMCs for the first time.

Some published studies have shown that microRNA-30a-3p is a suppressor gene in various tumor diseases, including liver cancer, esophageal cancer, gastric cancer, and other cancers [18]. MicroRNA-30a-3p has also been confirmed by some studies to be related to the pathological development of some diseases: asthma, osteoarthritis and rheumatoid arthritis [19]. However, we first discovered that microRNA-30a-3p was abnormally expressed in restenotic ASO vessels and suppressed VSMC proliferation and migration (Figures 1, 2). VSMC proliferation and migration are affected by their cell phenotype. VSMCs show different phenotypes: contractile and synthetic. Synthetic VSMCs show enhanced proliferation and migration, exist in neointima formation and participate in arteriosclerosis development [20]. However, microRNA-30a-3p can partially prevent the switch of contractile VSMCs into synthetic VSMCs (Figure 3). These studies suggest that microRNA-30a-3p can inhibit intimal hyperplasia by inhibiting VSMC proliferation, migration and phenotype switching. Of course, other mechanisms by which microRNA-30a-3p achieves its functions remain to be explored.

Through the prediction of target gene software and the classification and analysis of these target genes, we found that ROCK2 is probably a microRNA-30a-3p target. ROCK2 is a Rho kinase (ROCK1 and ROCK2) belonging to the Rho/ROCK signaling pathway. ROCK induces the reorganization of the actin cytoskeleton and is closely related to basic cell functions: adhesion, proliferation, migration, contraction and gene expression [10, 21]. The increased activity of ROCK mediates excessive contraction of VSMCs, inflammatory cell recruitment, endothelial dysfunction, and vascular remodeling [10]. ROCK has been proven to participate in VSMC proliferation, migration and phenotypic switching [12]. ROCK can stimulate VSMC proliferation by inducting ERK, cyclin D1 and PCNA [22]. The ROCK-JNK signaling pathway is associated with VSMC migration [23] and regulates VSMC phenotype switching and vascular remodeling [24]. In this experiment, we reconfirmed that ROCK2 can stimulate VSMC proliferation and migration (Figure 5). To confirm that microRNA-30a-3p functions through ROCK2, we performed a luciferase reporter assay (Figure 4). Then, ROCK2 was overexpressed by ROCK2-LV, and weakened the microRNA-30a-3p inhibitory effect on VSMC function (Figure 5). These studies showed that ROCK2 is a microRNA-30a-3p functional target in VSMCs.

VSMC overproliferation and migration are important for neointimal formation, and microRNAs participate in this process. In recent years, many microRNAs have been experimentally demonstrated to participate in VSMC function and the development of arteriosclerosis, including miR-302a, miR-4487, and miR-31-5p [25]. Because microRNA-30a-3p could obviously suppress VSMC function *in vitro*, we inferred that microRNA-30a-3p may influence the formation of neointima *in vivo*. The study indicated that microRNA-30a-3p was downregulated in the vessels injured by the balloon; however, exogenous LV-miR-30a-3p obviously upregulated microRNA-30a-3p expression in injured vessels and effectively inhibited neointima formation and VSMC proliferation (Figure 6).

In conclusion, microRNA-30a-3p suppressed VSMC proliferation, migration and phenotypic switching by targeting ROCK2. Moreover, exogenous microRNA-30a-3p inhibited neointima formation *in vivo*. Thus, strategies to increase microRNA-30a-3p content in tissues are likely to be an effective treatment for postoperative restenosis and ASO. Of course, more complete research is needed to confirm this hypothesis. Next, we will construct microRNA-30a-3p knockout or transgenic rats to further probe its function. Our work will be helpful for the treatment of cardiovascular diseases with noncoding RNAs. The introduction of microRNA into vascular tissue using lentivirus is used in animal experiments, but if it is to be used clinically, we need a safer and more efficient method that is more suitable for humans, which requires more research.

## Supplementary Material

Supplementary Figures

Supplementary Tables

## References

[1] Alloubani A, Nimer R, Samara R. Relationship between Hyperlipidemia, Cardiovascular Disease and Stroke: A Systematic Review. Curr Cardiol Rev. 2021; 17:e051121189015. 10.2174/1573403X16999201210200342 33305711 PMC8950504

[2] Kaur P, Kotru S, Singh S, Munshi A. miRNA signatures in diabetic retinopathy and nephropathy: delineating underlying mechanisms. J Physiol Biochem. 2022; 78:19–37. 10.1007/s13105-021-00867-0 35098434

[3] Lian W, Nie H, Yuan Y, Wang K, Chen W, Ding L. Clinical Significance of Endothelin-1 And C Reaction Protein in Restenosis After the Intervention of Lower Extremity Arteriosclerosis Obliterans. J Invest Surg. 2021; 34:765–70. 10.1080/08941939.2019.1690600 31996054

[4] Li S, Yu G, Jing F, Chen H, Liu A, Luo M, Huang W, Pu P, Chen M. RING finger protein 10 attenuates vascular restenosis by inhibiting vascular smooth muscle cell hyperproliferation *in vivo* and *vitro*. IUBMB Life. 2019; 71:632–42. 10.1002/iub.1995 30597731

[5] Robinson EL, Emanueli C, Martelli F, Devaux Y. Leveraging non-coding RNAs to fight cardiovascular disease: the EU-CardioRNA network. Eur Heart J. 2021; 42:4881–3. 10.1093/eurheartj/ehab326 34109376

[6] Vanhaverbeke M, Attard R, Bartekova M, Ben-Aicha S, Brandenburger T, de Gonzalo-Calvo D, Emanueli C, Farrugia R, Grillari J, Hackl M, Kalocayova B, Martelli F, Scholz M, et al. Peripheral blood RNA biomarkers for cardiovascular disease from bench to bedside: a position paper from the EU-CardioRNA COST action CA17129. Cardiovasc Res. 2022; 118:3183–97. 10.1093/cvr/cvab327 34648023 PMC9799060

[7] Wronska A. The Role of microRNA in the Development, Diagnosis, and Treatment of Cardiovascular Disease: Recent Developments. J Pharmacol Exp Ther. 2023; 384:123–32. 10.1124/jpet.121.001152 35779862

[8] Wang X, Li H, Zhang Y, Liu Q, Sun X, He X, Yang Q, Yuan P, Zhou X. Suppression of miR-4463 promotes phenotypic switching in VSMCs treated with Ox-LDL. Cell Tissue Res. 2021; 383:1155–65. 10.1007/s00441-020-03338-y 33245416

[9] Wu YT, Chen L, Tan ZB, Fan HJ, Xie LP, Zhang WT, Chen HM, Li J, Liu B, Zhou YC. Luteolin Inhibits Vascular Smooth Muscle Cell Proliferation and Migration by Inhibiting TGFBR1 Signaling. Front Pharmacol. 2018; 9:1059. 10.3389/fphar.2018.01059 30298006 PMC6160560

[10] Surma M, Wei L, Shi J. Rho kinase as a therapeutic target in cardiovascular disease. Future Cardiol. 2011; 7:657–71. 10.2217/fca.11.51 21929346 PMC3193795

[11] Wang Y, Zheng XR, Riddick N, Bryden M, Baur W, Zhang X, Surks HK. ROCK isoform regulation of myosin phosphatase and contractility in vascular smooth muscle cells. Circ Res. 2009; 104:531–40. 10.1161/CIRCRESAHA.108.188524 19131646 PMC2649695

[12] Lou L, Zheng W. Micro RNA 200a contributes to the smooth muscle cells growth in aged-related erectile dysfunction via regulating Rho/ROCK pathway. Andrologia. 2022; 54:e14503. 10.1111/and.14503 35778809

[13] Shamaki GR, Markson F, Soji-Ayoade D, Agwuegbo CC, Bamgbose MO, Tamunoinemi BM. Peripheral Artery Disease: A Comprehensive Updated Review. Curr Probl Cardiol. 2022; 47:101082. 10.1016/j.cpcardiol.2021.101082 34906615

[14] Beckman JA, Schneider PA, Conte MS. Advances in Revascularization for Peripheral Artery Disease: Revascularization in PAD. Circ Res. 2021; 128:1885–912. 10.1161/CIRCRESAHA.121.318261 34110904

[15] Samra M, Srivastava K. Non-coding RNA and their potential role in cardiovascular diseases. Gene. 2023; 851:147011. 10.1016/j.gene.2022.147011 36326502

[16] Hu W, Chang G, Zhang M, Li Y, Yin L, Huang Y, Feng C, Gu Y, Wen D, Wang S. MicroRNA-125a-3p affects smooth muscle cell function in vascular stenosis. J Mol Cell Cardiol. 2019; 136:85–94. 10.1016/j.yjmcc.2019.08.014 31499051

[17] Grootaert MO, Bennett MR. Vascular smooth muscle cells in atherosclerosis: time for a re-assessment. Cardiovasc Res. 2021; 117:2326–39. 10.1093/cvr/cvab046 33576407 PMC8479803

[18] Zhou K, Cao D, Wang Y, Wang L, Meng X. Hsa-miR-30a-3p attenuates gastric adenocarcinoma proliferation and metastasis via APBB2. Aging (Albany NY). 2021; 13:16763–72. 10.18632/aging.203197 34182542 PMC8266363

[19] Lv X, Huang J, Wang H. MiR-30a-3p ameliorates oxidative stress in rheumatoid arthritis synovial fibroblasts via activation of Nrf2-ARE signaling pathway. Immunol Lett. 2021; 232:1–8. 10.1016/j.imlet.2021.01.004 33450324

[20] Tang HY, Chen AQ, Zhang H, Gao XF, Kong XQ, Zhang JJ. Vascular Smooth Muscle Cells Phenotypic Switching in Cardiovascular Diseases. Cells. 2022; 11:4060. 10.3390/cells11244060 36552822 PMC9777337

[21] Porter L, Minaisah RM, Ahmed S, Ali S, Norton R, Zhang Q, Ferraro E, Molenaar C, Holt M, Cox S, Fountain S, Shanahan C, Warren D. SUN1/2 Are Essential for RhoA/ROCK-Regulated Actomyosin Activity in Isolated Vascular Smooth Muscle Cells. Cells. 2020; 9:132. 10.3390/cells9010132 31935926 PMC7017107

[22] Zhao Y, Lv M, Lin H, Cui Y, Wei X, Qin Y, Kohama K, Gao Y. Rho-associated protein kinase isoforms stimulate proliferation of vascular smooth muscle cells through ERK and induction of cyclin D1 and PCNA. Biochem Biophys Res Commun. 2013; 432:488–93. 10.1016/j.bbrc.2013.02.009 23402758

[23] Luo Z, Deng H, Fang Z, Zeng A, Chen Y, Zhang W, Lu Q. Ligustilide Inhibited Rat Vascular Smooth Muscle Cells Migration via c-Myc/MMP2 and ROCK/JNK Signaling Pathway. J Food Sci. 2019; 84:3573–83. 10.1111/1750-3841.14936 31762036

[24] Tang L, Dai F, Liu Y, Yu X, Huang C, Wang Y, Yao W. RhoA/ROCK signaling regulates smooth muscle phenotypic modulation and vascular remodeling via the JNK pathway and vimentin cytoskeleton. Pharmacol Res. 2018; 133:201–12. 10.1016/j.phrs.2018.05.011 29791873

[25] Zhou B, Wu N, Yan Y, Wu LL, Zhu GQ, Xiong XQ. Angiotensin II-induced miR-31-5p upregulation promotes vascular smooth muscle cell proliferation and migration. Exp Cell Res. 2022; 419:113303. 10.1016/j.yexcr.2022.113303 35934101

