# Supplementary material for: MicroRNA-30a-3p: a potential noncoding RNA target for the treatment of arteriosclerosis obliterans
Source: Aging (Albany NY). 2023 Oct 27;15(21):11875–90. doi: 10.18632/aging.205154 (PMC10683622; doi:10.18632/aging.205154)
Supplement: Supplementary Figures [file aging-15-205154-s001.pdf]

SUPPLEMENTARY FIGURES

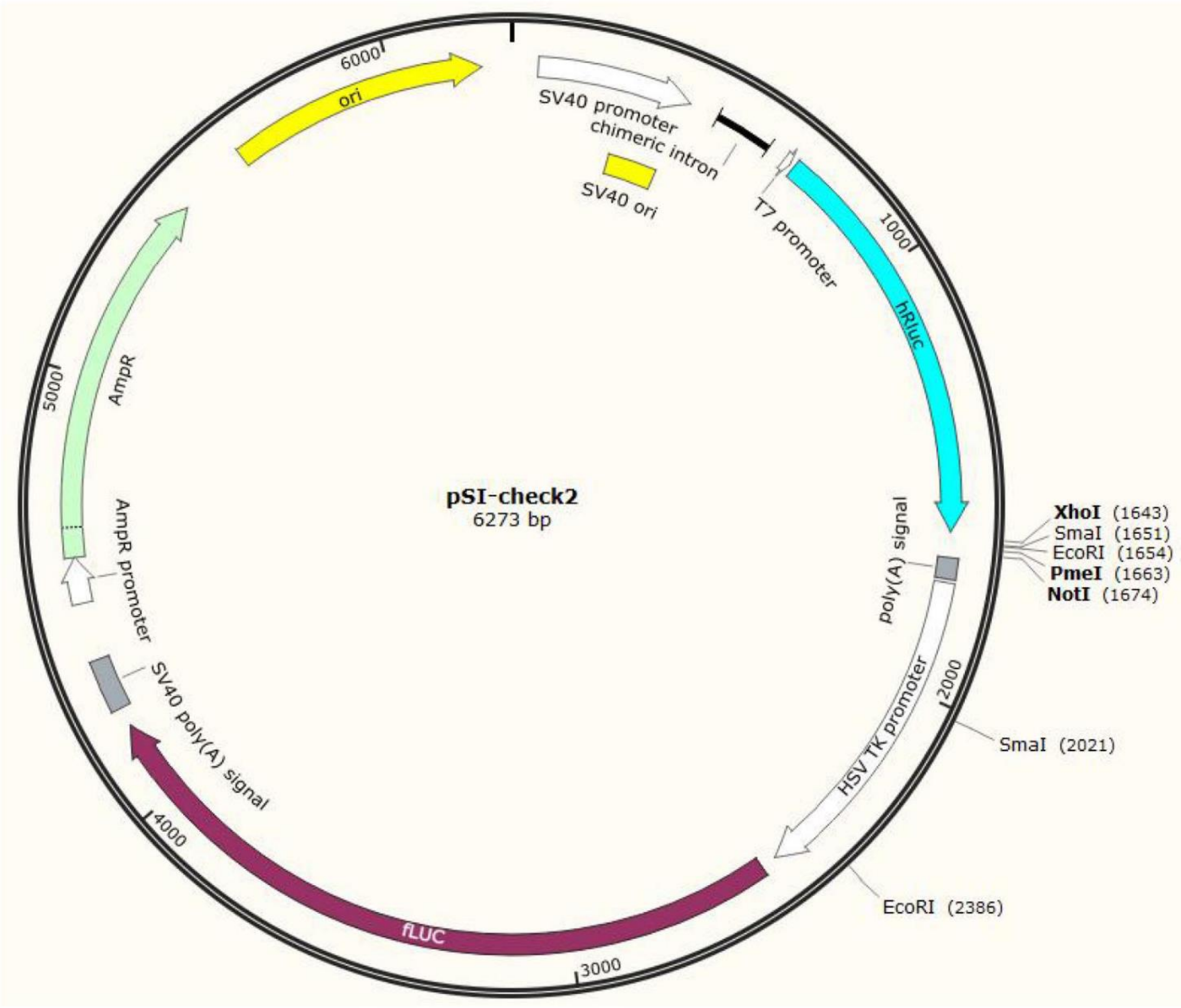

Supplementary Figure 1. The pSI-Check2 vector map.

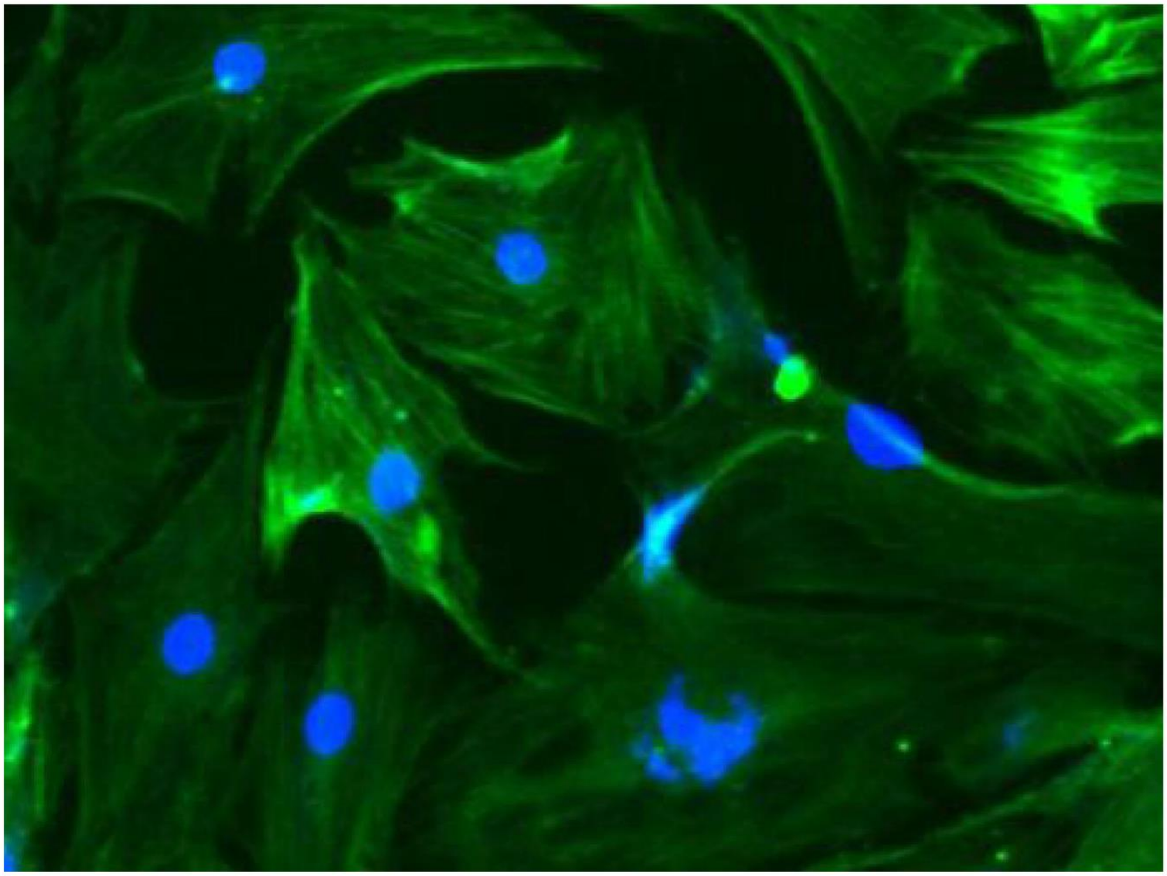

Supplementary Figure 2. Identification of primary cultured human artery smooth muscle cells. The green fluorescence indicates cells expressing SM-α-actin. DAPI-stained nuclei are shown in blue.
