# Supplementary material for: MicroRNA-30a-3p: a potential noncoding RNA target for the treatment of arteriosclerosis obliterans
Source: Aging (Albany NY). 2023 Oct 27;15(21):11875–90. doi: 10.18632/aging.205154 (PMC10683622; doi:10.18632/aging.205154)
Supplement: Supplementary Tables [file aging-15-205154-s002.pdf]

SUPPLEMENTARY TABLES

Supplementary Table 1. Patient information.

No.	Sex	Age	Diagnosis	Smoking (years)	Alcohol (years)	Hypertension (years)	T2DM/Blood glucose (mM)
1	M	62	ASO	—	25Y	10Y	—/8.1
2	M	53	ASO	30Y	—	25Y	—/8.0
3	M	50	ASO	—	20Y	16Y	10Y/N
4	M	67	ASO	—	—	29Y	—/N
5	M	73	ASO	30Y	30Y	25Y	20Y/9.2
6	M	77	ASO	45Y	55Y	17Y	10Y/10.1
7	F	73	ASO	40Y	14Y	—	—/N
8	M	77	ASO	—	—	—	—/7.1
9	M	45	Normal	—	—	—	—
10	M	42	Normal	—	—	—	—
11	M	59	Normal	—	—	—	—
12	M	30	Normal	—	—	—	—
13	F	43	Normal	—	—	—	—
14	M	47	Normal	—	—	—	—
15	F	29	Normal	—	—	—	—
16	M	32	Normal	—	—	—	—

ASO, arteriosclerosis obliterans; T2DM, type 2 diabetes; N, normal blood glucose levels.

Supplementary Table 2. Sequence of the probe for the *in-situ* hybridization.

Probe for ISH	5'Dig- GCTGCAAACATCCGACTGAAAG-3’

Supplementary Table 3. Sequence information of the target genes for luciferase reports.

LT-h-ROCK2-wt:

TCTATGAAAGCAGTCATTATTCAAGGTGATCGTATTCTTCCAGTGAAAACAAGACTGAAA
TATGATGGCCCAAAATTTATTAAAAAGCTATATTTTCCTGAGAGACTGATACATACACTCC
CATAATAGATACTATTGTGGAAACTGAAGAGTAGCTACTGGCCTACTGATAGACTGAAAC
TGGTTTTGTTATTCTTAACGTTCTCCAGTCTGCACTCTGCTGTGCTGTCTGTGCTCAGATA
TTTTTAATAAACTTTATTAAGGTGAAATAATTTCAAGTTTACATATGAGTAACTGAAATAT
TTGAGTAAAAAAGGCAAAAGTTTAAATTTGGAATGCTGTATATATTAGAGAATATGGTCT
TCTAGGATTCTGGTGGTAAAGTAGAGAAACTCGGACACTATATTAACCTTACTGAAACAT
GCTTTAAATCGTGTCTAAAATCTGATTTGAGCAAGTCACTGCCGAGCTTCTGAGTCTAAG
TAATGAACTGCATGGTTTAGAAAATTATAAATATTTGATCAGCTGTCCTCTCTGAAAAAA
ATTGCACCCCTTTTTCTTTTTTAAAATTTTGTTGCATCTTCGTAGTAATGTAATTGT

LT-h-ROCK2-mut:

TCTATGAAAGCAGTCATTATTCAAGGTGATCGTATTCTTCCAGTGAAAACAAGTCAGTAT
TATGATGGCCCAAAATTTATTAAAAAGCTATATTTTCCTGAGAGACTGATACATACACTCC
CATAATAGATACTATTGTGGAAACTGAAGAGTAGCTACTGGCCTACTGATAGTCAGTATC
TGGTTTTGTTATTCTTAACGTTCTCCAGTCTGCACTCTGCTGTGCTGTCTGTGCTCAGATA
TTTTTAATAAACTTTATTAAGGTGAAATAATTTCAAGTTTACATATGAGTATCAGTATTATT
TGAGTAAAAAAGGCAAAAGTTTAAATTTGGAATGCTGTATATATTAGAGAATATGGTCTT
CTAGGATTCTGGTGGTAAAGTAGAGAAACTCGGACACTATATTAACCTTTCAGTATCATG
CTTTAAATCGTGTCTAAAATCTGATTTGAGCAAGTCACTGCCGAGCTTCTGAGTCTAAGT
AATGAACTGCATGGTTTAGAAAATTATAAATATTTGATCAGCTGTCCTCTGTCATAAAAAT
TGCACCCCTTTTTCTTTTTTAAAATTTTGTTGCATCTTCGTAGTAATGTAATTGT
